# The Evolving Field of Acute Coronary Syndrome Management: A Critical Appraisal of the 2023 European Society of Cardiology Guidelines for the Management of Acute Coronary Syndrome

**DOI:** 10.3390/jcm13071885

**Published:** 2024-03-25

**Authors:** Roberto Licordari, Francesco Costa, Victoria Garcia-Ruiz, Mamas A. Mamas, Guillaume Marquis-Gravel, Jose M. de la Torre Hernandez, Juan Jose Gomez Doblas, Manuel Jimenez-Navarro, Jorge Rodriguez-Capitan, Cristobal Urbano-Carrillo, Luis Ortega-Paz, Raffaele Piccolo, Antonio Giovanni Versace, Gianluca Di Bella, Giuseppe Andò, Dominick J. Angiolillo, Marco Valgimigli, Antonio Micari

**Affiliations:** 1 Department of Biomedical and Dental Sciences and of Morphological and Functional Images, University of Messina, 98122 Messina, Italy; robertolicordari@gmail.com (R.L.); amicari@unime.it (A.M.); 2 Division of Cardiology, GIOMI Hospital, 98122 Messina, Italy; mavigaru@gmail.com; 3 Keele Cardiovascular Research Group, Keele University, Keele ST5 5BG, UK; m.mamas@keele.ac.uk; 4 Montréal Heart Institute, Faculty of Medicine, Université de Montréal, Montréal, QC H3T 1J4, Canada; guillaume.marquis.gravel@umontreal.ca; 5 Cardiology Department, Hospital Marqués de Valdecilla, IDIVAL, 39008 Santander, Spain; josemariadela.torre@scsalud.es; 6 Área del Corazón, Hospital Universitario Virgen de la Victoria, CIBERCV, IBIMA Plataforma BIONAND, Departamento de Medicina UMA, 29010 Malaga, Spain; jjgomezdoblas@gmail.com (J.J.G.D.); mjimeneznavarro@uma.es (M.J.-N.); capijorge@hotmail.com (J.R.-C.); 7 Servicio de Cardiología, Hospital Regional Universitario de Málaga, 29010 Málaga, Spain; cristobal.urbano.sspa@juntadeandalucia.es; 8 Division of Cardiology, University of Florida College of Medicine, Jacksonville, FL 32209, USAdominick.angiolillo@jax.ufl.edu (D.J.A.); 9 Department of Advanced Biomedical Sciences, University of Naples Federico II, 80138 Naples, Italy; raffaele.piccolo@unina.it; 10 Emergency Medicine Unit, Papardo Hospital, 98158 Messina, Italy; aversace@unime.it; 11 Department of Clinical and Experimental Medicine, University of Messina, 98122 Messina, Italy; dibellag@unime.it (G.D.B.); giuseppe.ando@unime.it (G.A.); 12 Cardiocentro Ticino Institute, Ente Ospedaliero Cantonale, 6500 Lugano, Switzerland; marco.valgimigli@eoc.ch

**Keywords:** acute coronary syndrome, clinical practice guidelines, dual antiplatelet therapy, intravascular imaging, colchicine, polypill

## Abstract

Acute coronary syndromes (ACS), encompassing conditions like ST-elevation myocardial infarction (STEMI) and non-ST-elevation acute coronary syndromes (NSTE-ACS), represent a significant challenge in cardiovascular care due to their complex pathophysiology and substantial impact on morbidity and mortality. The 2023 European Society of Cardiology (ESC) guidelines for ACS management introduce several updates in key areas such as invasive treatment timing in NSTE-ACS, pre-treatment strategies, approaches to multivessel disease, and the use of imaging modalities including computed tomography (CT) coronary angiography, magnetic resonance imaging (MRI), and intracoronary imaging techniques, such as optical coherence tomography (OCT) and intravascular ultrasound (IVUS). They also address a modulation of antiplatelet therapy, taking into consideration different patient risk profiles, and introduce new recommendations for low-dose colchicine. These guidelines provide important evidence-based updates in practice, reflecting an evolution in the understanding and management of ACS, yet some potentially missed opportunities for more personalized care and technology adoption are discussed.

## 1. Introduction

Acute coronary syndromes, encompassing a spectrum of conditions including ST-elevation myocardial infarction (STEMI) and non-ST-elevation acute coronary syndromes (NSTE-ACS), represent a critical area in cardiovascular medicine due to their prevalence, morbidity, and mortality. The 2023 European Society of Cardiology (ESC) guidelines for the management of acute coronary syndromes (ACS) provide a comprehensive framework that reflects the latest scientific insights and therapeutic advancements in this field [1]. These guidelines mark a significant evolution in the approach to ACS by consolidating the management of STEMI and NSTE-ACS into a unified framework. In this review, we aim to discuss key aspects that, in our opinion, constitute the main novelties of the current edition of the ACS ESC guidelines, critically appraising some potentially missed opportunities.

## 2. Condensing STEMI and NSTE-ACS

The decision to condense the management of STEMI and NSTE-ACS into a single document is a testament to the evolving understanding of these conditions as more of a continuum rather than distinct entities. The delineation between STEMI and NSTE-ACS has traditionally been a subject of clinical debate, often leading to a dichotomous approach to management. However, this division has, at times, been more reflective of an artificial classification rather than a true difference in pathophysiology or clinical outcome. Both STEMI and NSTE-ACS represent different manifestations of the same underlying atherosclerotic process, characterized by plaque rupture or erosion and subsequent coronary thrombosis. This shared pathophysiology extends to their risk factors, clinical implications, and the post-acute management, suggesting a more integrated approach could improve patient outcomes. By consolidating these conditions, the guidelines seek to reduce the ambiguity and artificiality inherent in past classifications [2,3], fostering a more nuanced understanding and treatment of the disease spectrum. In addition, from a scientific society point of view, a common framework within the same document allows more consistent recommendations, at difference with past experiences in which temporal- and committee-driven differences in evidence interpretation have created inconsistencies among different guideline editions.

## 3. Invasive Treatment in Acute Coronary Syndromes without ST-Segment Elevation

In the domain of STEMI, primary coronary intervention (PCI) recommendations and timing retain their status quo from prior editions [2,4]. The execution of primary PCI for patients with symptom onset under 12 h continues to be highly advocated, with a target of 60 min for presentations at 24/7 PCI centers and 90 min at non-PCI centers [1]. The innovation in the current guidelines primarily lies in the timing of invasive treatment for NSTE-ACS. The guidelines now advocate for an early invasive strategy (within 24 h) for patients with a confirmed diagnosis, dynamic ST-segment changes, transient ST-segment elevation, or a GRACE risk score above 140, marking a shift to a class IIa level of evidence A [1]. Previously, such an approach for these high-risk patients was recommended with a class I level of evidence A [3] (Table 1). This adjustment stems from real-world application challenges, wherein directing most patients with NSTE-ACS to PCI centers very early is logistically complex. Moreover, it is crucial to acknowledge that in many cases, NSTEMIs are often associated with comorbidities such as infection, sepsis, or cancer. These conditions may necessitate delayed or modified approaches to invasive treatment, reflecting a comprehensive understanding of patient care beyond the acute coronary event itself. In addition, no study has conclusively proven the superiority of an early invasive strategy over routine invasive strategies for NSTE-ACS. A recent meta-analysis by Kite et al., comprising 17 randomized clinical trials with over 10,000 patients, showed that early invasive treatment reduces the risk of recurrent ischemia and rehospitalizations, with no additional benefit for hard clinical endpoints (all-cause mortality, admission for HF, repeat re-vascularization, major bleeding and stroke) [5]. We agree that, considering the shaky grounds of evidence in this field, allowing clinicians a higher level of flexibility in managing the logistics of these patients could be useful, also considering the potential legal impact of too strict recommendations that might not be feasible in all contexts and situations. Nevertheless, current guidelines highlight the need to adjust this context and improve current standards of care throughout Europe.

## 4. Pretreatment

Pretreatment refers to a strategy whereby an antiplatelet drug, usually an oral P2Y12 receptor inhibitor, is administered before coronary angiography and thus before the knowledge of the coronary anatomy. The lack of definitive evidence in favor of pretreatment from randomized controlled trials has triggered in NSTE-ACS a downgrade of this practice, since 2020. The current guidelines also downgrade pretreatment in STEMI patients [1,3] (from class I level of evidence A to the current class IIb level of evidence B) [1,2] based on a re-interpretation of the ATLANTIC trial (Administration of Ticagrelor in the Cath Lab or in the Ambulance for New ST-Elevation Myocardial Infarction to Open the Coronary Artery) [6], which was published 10 years ago and had been much more favorably interpreted by prior guidelines’ committees (Table 1). The ATLANTIC trial, which had a mechanistic endpoint of ST-segment improvement in the post-procedural ECG or TIMI flow in coronary angiography, randomized patients with STEMI to a loading dose of ticagrelor administered before the procedure or at the time of primary PCI with a time difference between the two treatments of only 31 min—an extremely short time, especially in the context of STEMI, for any oral antiplatelet drug to exert its benefit [6]. Furthermore, in the ATLANTIC study, a routine pre-treatment strategy did not significantly increase the frequency of major or minor bleeding compared to the administration of the ticagrelor loading dose in the Cath-lab [6].

Redfors et al. used data from the Swedish Coronary Angiography and Angioplasty Registry to study the possible benefits of pretreatment in STEMI patients; 44,804 patients were included in the study. Pretreatment did not show a benefit in terms of survival at 30 days, reduced IRA occlusion at 30 days, decreased stent thrombosis or higher risk of in-hospital bleeding [7].

In addition, a recent study by Rohla et al., published subsequent to the release of the guidelines, investigated 1963 STEMI patients undergoing PCI within the Bern-PCI registry (2016–2020) to assess the outcomes of immediate versus delayed P2Y12 inhibitor administration. This cohort analysis divided patients based on the timing of P2Y12 inhibitor receipt—either immediate or post-coronary anatomy confirmation. The primary endpoint, 30-day MACCEs (encompassing all-cause mortality, recurrent myocardial infarction, stroke, or definitive stent thrombosis), showed no significant difference between the groups [8].

Hence, while acknowledging the limitations of these analyses, it should be emphasized that there is no compelling evidence that early pretreatment in STEMI is effective. This is in line with the great advancements made in STEMI networks allowing prompt primary PCI after diagnosis in most patients, which limit the potential benefits of pretreatment in the pre-hospital phase. In addition, pretreatment is generally administered in a challenging environment, often in an ambulance and by non-specialized caregivers with limited access to patient history and cardiac imaging (e.g., echocardiography to rule out aortic dissection). Hence, the guidelines’ recommendation to avoid routine pretreatment in STEMI seems reasonable. It should be noted that in a situation with a longer delay to the Cath-lab, pre-treatment administration could provide a potential benefit, justifying the current recommendation. On the contrary, the potential advantage of pre-treatment, as seen in a deeper evaluation of the ATLANTIC trial [9], might be mitigated by the introduction of modern stent platforms and novel parenteral pharmacologic strategies that may close this gap.

In contrast, for patients with NSTE-ACS, the landscape is more nuanced considering the variable delay to angiography. In the previous guidelines, routine pre-treatment in patients receiving an early invasive strategy (within 24 h) was not recommended, in line with the current lack of evidence from randomized trials that a pretreatment is effective.

In this setting, the ACCOAST trial explored the effects of early prasugrel administration in NSTE-ACS patients. In this study, over 4000 patients were randomized to receive either a 30 mg loading dose of prasugrel prior to angiography or a placebo. The trial’s primary efficacy endpoint, comprising cardiovascular death, MI, stroke, urgent revascularization, or glycoprotein IIb/IIIa inhibitor rescue therapy, showed no significant difference between the two groups within seven days. However, a notable increase in major bleeding episodes, as defined by the TIMI criteria, was observed in the pre-treatment group, including a threefold rise in non-CABG-related major bleeding and a sixfold increase in life-threatening bleeding [10].

Hence, in line with this evidence and in continuity with prior recommendations from the 2020 NSTE-ACS guidelines, routine pretreatment remains contraindicated by the current document. However, a class IIb recommendation for pretreatment is present for those that are expected to undergo an invasive management later than 24 h after the initial diagnosis, which is still a reflection of current practice in certain geographical areas [11]. The evidence suggesting a lack of benefit from pretreatment was derived from trials where the interval between pretreatment and angiography was notably brief, thereby not ruling out the potential for benefits in cases of longer delays. Again, we believe that providing flexibility to clinicians in this setting is reasonable.

## 5. Multivessel Treatment Strategies

About half of the patients with ACS present multivessel disease [12]. The management of the non-culprit lesion varies according to the clinical context and the type of ACS. In the last decade, several randomized clinical trials have demonstrated the benefit of the preventive revascularization of non-culprit lesions in patients with STEMI after successful primary PCI of the culprit lesion [13,14]. A recent systematic review of 10 randomized clinical trials that included more than 7000 patients with STEMI and multivessel disease found that complete revascularization was associated with a reduction in cardiovascular mortality and the composite of cardiovascular death and myocardial reinfarction compared to PCI of the culprit lesion alone [15]. In light of all this, the recent guidelines have attributed a recommendation of a class I level of evidence A (in the previous edition it was class IIa level of evidence A) to complete revascularization in patients with multivessel STEMI during the index procedure, or within 45 days of the same [1]; in the previous edition, instead, the staged procedure had to be performed before the discharge of the index hospitalization [2] (Table 1). This recommendation seems to contradict the findings of the recent MULTISTARS-AMI trial, which included 840 STEMI patients. These participants were randomized into two groups: one underwent PCI on non-culprit lesions during the index procedure, while the other had PCI on non-culprit lesions within 19–45 days later. The study’s primary outcome, a composite of mortality, nonfatal MI, stroke, unexpected ischemia-driven target lesion revascularization (ID-TLR), and hospital admissions due to heart failure after one year, showed a significant difference between immediate and staged PCI. It is important to highlight that the timing of staged PCI in this study was longer than in usual practice (which typically happens before discharge). It should also be noted that the publication of the MULTISTARS trial occurred after the development of the guidelines [16]. However, challenges in the adjudication of peri-procedural MI in the context of STEMI might limit the ability of such trial design to provide a definite answer on this matter. Published simultaneously with guidelines and consistently with them, the FIRE trial demonstrated that elderly patients with multivessel disease undergoing physiology-guided complete revascularization had a lower risk of the composite ischemic endpoint (a composite of death, myocardial infarction, stroke, or any revascularization) at 1 year than those who received culprit-lesion-only PCI [17].

A notable exception of this paradigm is the setting of ACS and cardiogenic shock. In this setting, multivessel coronary disease is present in about 80% of cases [18]. In the CULPRIT-SHOCK trial (Culprit Lesion Only PCI versus Multivessel PCI in Cardiogenic Shock) that included patients with ACS, PCI of the culprit lesion alone during the index procedure was associated with a significant reduction in all-cause mortality at 30 days and 1 year follow-up compared to immediate complete revascularization [19]. Consequently, the guidelines recommend, with class I, the PCI of the culprit lesion alone in patients with cardiogenic shock and multivessel disease, leaving the possibility of completing revascularization in a deferred procedure, with a class IIa level of evidence C (Table 1) [1]. In this setting the use of Mechanical Circulatory Support (MCS) devices remains debated; however, the current guidelines suggest a class IIa level of evidence C for the use of Intra-Aortic Balloon Pump (IABP) in cases of hemodynamic instability or cardiogenic shock. This recommendation draws on non-randomized studies, such as a propensity-matched registry analysis, indicating that micro-axial MCS support might lead to more complications and higher mortality compared to IABP [20]. On the contrary, the IABP-SHOCK II trial further demonstrated that the routine use of IABP in ACS and CS patients does not reduce mortality at 30 days, 1 year, or 6 years [21].

## 6. Anatomy vs. Physiology for Non-Culprit Lesions

The ACS guidelines address the management of multivessel disease in several settings, like hemodynamically stable STEMI patients undergoing primary PCI. In this context, PCI of non-infarct-related arteries (non-IRAs) should be guided by the angiographic severity, with a Class I recommendation and a level of evidence B [1] (Table 1). This recommendation is influenced by various studies that shed light on the comparative effectiveness of anatomical versus physiological approaches for non-culprit lesions. A meta-analysis by Wald et al., involving 10 randomized controlled trials with about 3000 STEMI patients undergoing PCI, assessed the outcomes of complete revascularization against IRA-only PCI. It found that the preventive PCI of the non-IRA led to a significant reduction in cardiac death and non-fatal myocardial infarction when decisions were based solely on angiographic severity [22]. This finding is supported by another meta-analysis by Gallone et al., reinforcing the potential value of an anatomically guided approach in this specific patient setting [23].

The FLOWER-MI Study aimed to determine whether FFR-guided strategy was superior to the angiography-guided procedures in reducing the composite primary outcome of death, nonfatal myocardial infarction, or unplanned hospitalization leading to urgent revascularization at one year. The primary outcome occurred in 5.5% of the FFR-guided group and 4.2% of the angiography-guided group, with no significant difference between the two strategies, concluding that an FFR-guided strategy did not offer significant benefits over an angiography-guided strategy [24].

In contrast, the FRAME AMI trial studied a physiology-driven strategy for managing non-infarct related artery (non-IRA) lesions in stable STEMI patients by comparing outcomes between FFR-guided PCI (performed if FFR ≤ 0.80) and conventional angiography-guided PCI (performed in case of >50% stenosis). The authors reported a reduced incidence of the composite primary endpoint of death, myocardial infarction, or repeat revascularization over a median observation period of 3.5 years. Nonetheless, it is crucial to acknowledge that this trial was concluded earlier than anticipated, and enrolled a smaller cohort than originally targeted [25].

While the available evidence may provide some nuances, current guidelines support angiography-based decisions as the primary tool to guide complete revascularization in STEMI patients. Other technologies that are not currently mentioned in the guidelines might be important in the future.

## 7. Management of Cardiac Arrest

The management of patients who have experienced a cardiac arrest complicating an ACS, particularly in the case of out-of-hospital cardiac arrest (OHCA), requires careful consideration. The guidelines delineate a tailored approach to these patients, emphasizing the importance of ECG findings in guiding subsequent interventions.

For patients with resuscitated cardiac arrest and persistent ST-segment elevation (or equivalents) on ECG, a primary percutaneous coronary intervention (PPCI) strategy is strongly recommended (Class I, Level of Evidence B) [1]. This recommendation is based on the critical need for timely revascularization in the presence of occlusive coronary artery disease, which is often the underlying cause of the cardiac arrest.

Conversely, the guidelines advise caution with routine immediate angiography in hemodynamically stable patients without persistent ST-segment elevation following resuscitated cardiac arrest (Class III, Level of Evidence A) [1]. This recommendation stems from a comprehensive evaluation of the evidence, including the COACT [26] and TOMAHAWK [27] randomized controlled trials (RCTs), which demonstrated that routine immediate ICA does not confer superiority over a delayed strategy based on clinical assessment.

## 8. Role of Imaging

Imaging, particularly non-invasive modalities, remains indispensable in evaluating ACS. Recent guidelines have nuanced the role of computed tomography coronary angiography and exercise testing, especially for patients presenting with ambiguous ACS symptoms without definitive ECG or troponin markers. While previously held in higher esteem (class I level of evidence B), these methods now carry a class IIa level of evidence A, reflecting a more calibrated use in the diagnostic workflow [1]. For cases of myocardial infarction with non-obstructive coronary arteries (MINOCA), cardiac magnetic resonance (CMR) has taken a prominent role, recommended post-angiography when the diagnosis remains uncertain (class I level of evidence B) (Table 1) [1]. Implementing CMR in this setting may be extremely useful and clarify life-threatening conditions. Moreover, left ventricular thrombus formation, although decreasing in incidence, is reported in up to 9% of patients following anterior STEMI. Although, echocardiography remains the first-line diagnostic tool, CMR now is recommended for ambiguous cases (class IIa level of recommendation C) [1].

Intracoronary imaging, notably optical coherence tomography (OCT) and intravascular ultrasound (IVUS), has significantly advanced the precision possible in diagnosing and managing ACS. These techniques are particularly valuable in identifying and characterizing ambiguous or culprit lesions in patients with non-obstructive coronary disease, suggesting their broader application in complex ACS cases. Consequently, current guidelines endorse intravascular imaging, especially OCT, as a preferred technique for clarifying atherothrombotic causes of ACS (class IIb level of evidence C) (Table 1) [1]. Further, intracoronary imaging is recommended for optimizing PCI outcomes, reflecting a class IIa level of evidence A [1]. In fact, given the abundance of data from multiple randomized controlled trials and meta-analyses supporting better clinical outcomes with imaging-guided PCI, especially through IVUS, we believe that not providing a higher class of recommendation for this strategy might be considered as a missed opportunity in this document.

The recent RENOVATE-COMPLEX-PCI trial randomized 1639 patients undergoing complex PCI, to either intravascular imaging-guided PCI (IVUS or OCT) or angiography-guided PCI. After a median follow up of 2.1, years the primary endpoint (a composite of death from cardiac causes, target-vessel-related myocardial infarction, or clinically driven target-vessel revascularization) was less frequently met in the guided PCI group, with no difference in procedure-related safety events [28]. In addition, the 2023 ESC Congress spotlighted several significant studies in intracoronary imaging, which, although not yet incorporated into the guidelines, signal a pivotal shift in PCI practice. Notable among these are ILUMIEN IV: OPTIMAL PCI [29], comparing OCT-guided coronary stent implantation with traditional angiography; OCTOBER [30], examining OCT vs. angiography in complex bifurcation lesions; and OCTIVUS [31], contrasting OCT-guided with IVUS-guided PCI in complex interventions. These studies collectively highlight the nuanced benefits and applications of intracoronary imaging techniques, advocating for their broader integration into clinical practice. A network meta-analysis, not yet published, was presented at the same congress, including ILUMIEN IV and OCTOBER trials with prior studies. This analysis, encompassing 20 randomized trials of intravascular imaging-guided PCI compared with angiography-guided PCI, included 12,428 patients, with both chronic and acute coronary syndromes. The primary endpoint was target lesion failure, defined as a composite of cardiac death, target vessel myocardial infarction, or target lesion revascularization. The results were compelling, showing a 31% reduction in the primary composite outcome of target lesion failure with intravascular imaging guidance compared to angiography. Secondary outcomes further bolstered the utility of imaging, demonstrating significant reductions in cardiac death, target vessel myocardial infarction, target lesion revascularization, and stent thrombosis. While the efficacy was consistent whether OCT or IVUS was used, it should be emphasized that IVUS-guided PCI is the strategy that offers the most evidence in its favor, and appears more practical when used to guide most procedures.

Historically, there was a call for more robust evidence to support the widespread adoption of these advanced diagnostic techniques. While the guidelines have cautiously assigned a class IIa recommendation of intracoronary imaging, the wealth of data presented at the ESC Congress and subsequent studies now furnish the clinical community with compelling evidence. This recent influx of data demonstrates significant improvements in patient outcomes when intracoronary imaging guides PCI. As such, it is time for the scientific community to acknowledge and embrace the consistent and convincing evidence favoring the routine use of intracoronary imaging.

## 9. Antiplatelet Therapy

The current guidelines recommend dual antiplatelet therapy (DAPT) as the default strategy for 12 months, regardless of the implanted stent, unless contraindicated (class I, level of evidence A) [1]. In specific clinical scenarios, the duration of DAPT may be shortened or extended.

In High-Bleeding-Risk (HBR) patients (e.g., based on Academic Research Consortium (ARC)-HBR criteria or PRECISE-DAPT ≥ 25), a 1-month DAPT followed by single antiplatelet therapy with aspirin or a P2Y12 inhibitor (class IIb, level of evidence B) is possible.

For HBR or non-HBR patients, who are not at high ischemic risk, DAPT can be prescribed for 3 or 6 months and then continued with aspirin or a P2Y12 inhibitor (class IIa, level of evidence A).

Switching from potent P2Y12 inhibitors to clopidogrel may be considered at one month (Class IIb, level of evidence A), while earlier de-escalation is contraindicated (Class III, level of evidence B) [1].

This approach considers the balance between minimizing bleeding risks while maintaining protection against thrombotic events.

The 6-month DAPT duration followed by aspirin was investigated in the SMART-DATE trial, which randomized roughly 2700 ACS patients to 6-month or at least 1-year (median duration 18 months) DAPT. The 6-month DAPT arm was associated with an increase in non-fatal ischemic events (myocardial infarction) from the moment of DAPT cessation, with no significant differences in bleeding outcomes [32].

Further, 1- to 3-month DAPT, followed by P2Y12 inhibitor (mostly ticagrelor and clopidogrel), was examined in the SIDNEY-2 meta-analysis, including over 24,000 patients undergoing PCI, comparing 1–3 months of DAPT followed by P2Y12i monotherapy (mostly clopidogrel or ticagrelor) versus continued DAPT. The short DAPT plus single P2Y12i arm did not show increased ischemic events (MI, death, or stroke) and halved the bleeding events (BARC 3 or 5), both in HBR and non-HBR patients [33]. Given the large body of evidence supporting a short DAPT (1–3 months), doubts arise about the similar class of recommendation for the 1- (IIb), 3-, and 6-month (IIa) DAPT approaches.

The SIDNEY-2 meta-analysis compared clopidogrel vs. ticagrelor in the experimental arm versus DAPT, and showed that ticagrelor, but not clopidogrel, was associated with a reduction in mortality [33]. The STOP-DAPT 2 ACS trial enrolled over 4100 patients undergoing PCI for ACS, randomizing them to 1 or 2 months of DAPT followed by clopidogrel monotherapy versus 12 months of DAPT with aspirin and clopidogrel. The short DAPT followed by clopidogrel monotherapy did not attain non-inferiority for the primary endpoint (composite of cardiovascular death, MI, stroke, or definite stent thrombosis) [34].

On the contrary, the T-PASS trial studied the effectiveness of ticagrelor monotherapy following less than 1 month (16 days) of DAPT versus a conventional 12-month ticagrelor-based DAPT in patients with ACS undergoing a bioresorbable polymer sirolimus-eluting stent (BP-SES) implantation. Here, 2850 patients were randomly assigned to short-term DAPT followed by ticagrelor monotherapy or a full 12-month course of ticagrelor-based DAPT. The primary endpoint was a composite of adverse events including all-cause death, myocardial infarction, stent thrombosis, stroke, and major bleeding at one-year post-procedure. The trial found that short-term DAPT followed by ticagrelor monotherapy was both noninferior and superior to the longer DAPT regimen, primarily driven by a significant reduction in major bleeding events [35]. These results raise doubts about generically referring to any P2Y12i after the DAPT period, given the different pharmacokintetic profiles and efficacies in large trials (e.g., ticagrelor and clopidogrel).

For HBR patients with ACS, a short-duration DAPT (1 month) strategy followed by SAPT (mainly clopidogrel) was studied in the MASTER-DAPT, enrolling over 4400 patients. This randomized trial demonstrated the non-inferiority of 1 month of DAPT versus DAPT for at least 3 months for net adverse clinical events (mortality, MI, stroke, major bleeding) and major adverse cardiovascular events (mortality, MI, or stroke) [36]. Moreover, a recent large meta-analysis including 11 trials with a total of 9006 HBR patients studied the efficacy and safety of an abbreviated DAPT (1 or 3 months). The abbreviated DAPT reduced major or clinically relevant non-major bleeding and cardiovascular mortality compared with standard DAPT. No difference in all-cause mortality, major adverse cardiovascular events, myocardial infarction, or stent thrombosis was observed [37].

Furthermore, antiplatelet therapy in patients with an indication to long-term oral anticoagulation, which are, per se, at HBR, is a point of concern [38]. A companion meta-analysis focused on this type of patients confirmed that a lower number of antithrombotic drugs is required to optimize outcomes in this group of patients [39]. Despite these findings, 1-month DAPT followed by aspirin monotherapy in HBR patients is recommended at class IIB.

Regarding DAPT de-escalation by switching the P2Y12i or by dose reduction, the rationale is to maintain dual antithrombotic drugs while reducing their potency and bleeding risk, switching after 1 month from aspirin + potent P2Y12 inhibitor to aspirin and clopidogrel. In some studies, this involved a dose reduction of ticagrelor (from 90 to 60 mg bid) and prasugrel (from 10 to 5 mg daily). The efficacy of the de-escalation strategy was demonstrated in a meta-analysis of four trials, including over 10,000 patients, comparing it to standard DAPT, with significant reductions in both ischemic and bleeding events [40]. These results were confirmed by another meta-analysis by Tavenier et al., which showed significant reductions in bleeding and major adverse cardiac events in patients assigned to DAPT de-escalation versus standard DAPT [41]. Given these findings, available at the time of guideline drafting and publication, one might consider whether the DAPT de-escalation strategy deserves more consideration.

In conclusion, advocating a 12-month DAPT regimen as default strategy, inherited from previous guideline iterations and not supported by current evidence, represents a significant missed opportunity [2,3,42]. This approach fails to further highlight the advantages of a personalized treatment, pushing back accumulated evidence derived from many years of focused research in this field [43].

## 10. Antiplatelet Therapy in Elderly Patients

The current guidelines offer a class IIb, level B evidence recommendation for the use of clopidogrel in elderly patients with ACS, particularly those identified at HBR. This guidance acknowledges clopidogrel’s varying and sometimes less potent effect on platelet inhibition. It suggests selecting clopidogrel when prasugrel or ticagrelor are either contraindicated or unavailable, or specifically for patients at HBR. Additionally, it is recommended to consider clopidogrel for patients who are 70 years of age or older.

This age demarcation at 70 years, while somewhat lower than the 75 years typically used in HBR definitions, derives from the inclusion criteria used in the POPULAR AGE study, which randomized roughly 1000 NSTE-ACS patients to receive DAPT with clopidogrel or ticagrelor/prasugrel. Designed for a non-inferiority endpoint of net adverse clinical events (NACE)—including all-cause death, myocardial infarction, stroke, and PLATO major and minor bleeding (also incorporating BARC 2)—the study reached the non-inferiority margin, with a reduction in minor bleedings with clopidogrel, albeit with a noted, but not statistically significant, increase in stent thrombosis (5 in clopidogrel arm vs 0 in ticagrelor one) [44].

These results are in contrast with those of the larger PLATO, which demonstrated the superiority of ticagrelor over clopidogrel for the primary ischemic endpoint, with no heterogeneity among different age subgroups [45]. Hence, considering the smaller population included in the POPULAR AGE trial, its non-inferiority design, and the lack of a formal inclusion of HBR patients, this new recommendation appears debatable.

## 11. Colchicine

The role of inflammation in atherosclerosis and acute coronary events is well-established. The current guidelines have introduced a class IIb, level A recommendation for the use of low-dose colchicine, an anti-inflammatory agent, reflecting its emerging role in cardiovascular secondary prevention (Table 1) [1].

The role of anti-inflammatory strategies was first explored in the CANTOS trial [46]. This pivotal study assessed the impact of canakinumab, a monoclonal antibody targeting interleukin-1β, in over 10,000 patients with a history of myocardial infarction. It compared three different dosage regimens against a placebo. Notably, canakinumab, particularly the 150 mg dose, demonstrated a reduced rate of the primary endpoint, which included nonfatal myocardial infarction, nonfatal stroke, and cardiovascular death. However, a higher incidence of fatal infections was observed in the canakinumab groups, though there was no significant difference in all-cause mortality. Despite these findings, the current clinical consideration of canakinumab remains limited, primarily due to its substantial cost.

The Colchicine Cardiovascular Outcomes Trial (COLCOT) is a pivotal study in this context. Enrolling about 4700 patients with recent ACS, COLCOT explored the efficacy of low-dose colchicine (0.5 mg daily). The results were compelling, showing a significant reduction in the primary composite endpoint, which included cardiovascular death, resuscitated cardiac arrest, myocardial infarction, stroke, or urgent revascularization, compared to placebo. Notably, an increased incidence of pneumonia was observed in the colchicine group, indicating a need for careful patient selection and monitoring [47]. Interestingly, this approach may be useful also remotely from a first episode of ACS. The Low-dose Colchicine trial-2 (LoDoCo2) included about 5500 patients with chronic coronary syndromes (CCS), 84% of whom had a history of ACS. This trial also administered low-dose colchicine (0.5 mg daily) and demonstrated a significant reduction in the primary endpoint, a composite of cardiovascular death, myocardial infarction, stroke, and ischemia-driven coronary revascularization. However, an increase in non-cardiovascular death was noted in the colchicine group, adding another layer of consideration in its application [48].

Both COLCOT and LoDoCo2 trials highlight the consistent benefits of colchicine in reducing cardiovascular events, irrespective of the history and timing of prior ACS. This recommendation marks a step forward in integrating inflammation-targeted therapy in cardiovascular care, and represents a potential option to consider in certain patients after evaluating the risk–benefit profile carefully. However, further research is needed to better understand a possible trade-off between a reduction in cardiovascular events and an increase in infections, which may increase non-cardiovascular morality.

## 12. Polypill

The concept of a polypill strategy involves the combination of multiple guideline-recommended treatments into a single pill, aimed at enhancing adherence and simplifying the treatment regimen for patients post-ACS. The current guidelines have introduced the polypill strategy as an option to improve adherence and outcomes in secondary prevention with a class IIa recommendation (Table 1) [1].

The rationale behind the polypill strategy is rooted in the challenge of medication adherence, which is crucial in preventing recurrent cardiovascular events. Adherence to medication post-ACS is often sub-optimal, with rates ranging from 50% in primary prevention to 66% in secondary prevention. The Secondary Prevention of Cardiovascular Disease in the Elderly (SECURE) study, the only randomized–controlled trial (RCT) testing this approach, enrolled almost 2500 patients with myocardial infarction within the previous 6 months. Patients were randomized to a polypill strategy (containing aspirin, ramipril, and atorvastatin) versus usual care. The primary composite outcome (cardiovascular death, nonfatal type 1 myocardial infarction, nonfatal ischemic stroke, or urgent revascularization) and the key secondary outcome (a composite of cardiovascular death, nonfatal type 1 myocardial infarction, or nonfatal ischemic stroke) were significantly lower in the polypill group. This was driven by a notable 33% reduction in cardiovascular mortality. Moreover, adherence to treatment was higher in the polypill group [49]. The polypill strategy represents a positive step forward in the management of ACS patients. It simplifies treatment regimens, enhances adherence, and, most importantly, has the potential to reduce cardiovascular events and mortality. This approach reflects a shift towards more patient-centered care, emphasizing the importance of both patient education and the role of healthcare professionals in supporting adherence. Yet, it will be important to see how this shift in recommendation will be backed up by the commercial availability of these combinations, as well as the availability of higher drug dosages for patients at the highest risk.

## 13. Final Considerations

**Figure 1:**
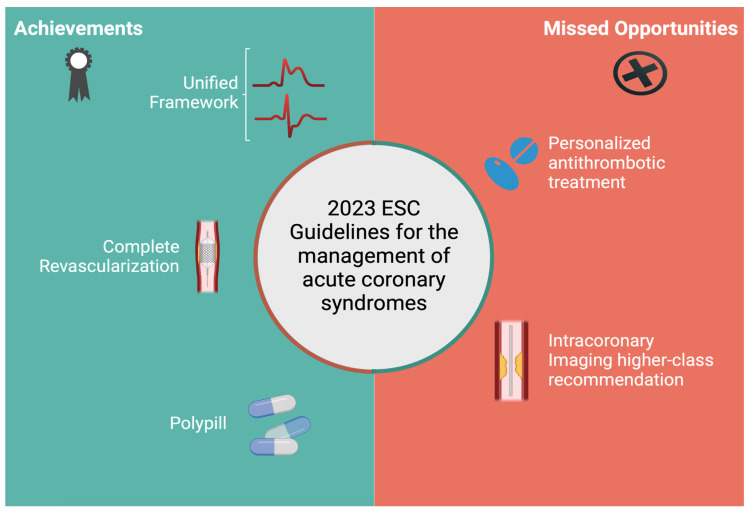
Summary of possible achievements and missed opportunities of the 2023 ESC guidelines for the management of Acute Coronary Syndromes.

The updated guidelines for ACS management represent a mixed landscape of advancement and caution (Figure 1). On one hand, they incorporate new recommendations, such as complete revascularization in STEMI, low-dose colchicine and polypills, highlighting a commitment to evidence-based advancements. On the other, while granting clinicians with a higher level of flexibility, especially in less advantaged settings, the push back of a more personalized approach to antithrombotic therapy appears questionable. These guidelines mark a clear step forward in some areas, but they also reflect the complexity and constant evolution inherent to cardiovascular care.

## Figures and Tables

**Table 1:** Comparative overview and key changes in the 2023 ESC Guidelines for Acute Coronary Syndromes.

Topic	Previous ACS Guidelines	2023 ACS Guidelines	Significance of Change
**Invasive Treatment in NSTE-ACS**	An early invasive strategy within 24 h is recommended in patients with any of the following high-risk criteria: Diagnosis of NSTEMI suggested by the diagnostic algorithm recommended in Section 3 Dynamic or presumably new contiguous ST/T-segment changes suggesting ongoing ischaemia Transient ST-segment elevation GRACE risk score > 140 (Class I LoE A) [3]	An early invasive strategy within 24 h should be considered in patients with at least one of the following high-risk criteria: Confirmed diagnosis of NSTEMI based on current recommended ESC hs-cTn algorithms Dynamic ST-segment or T wave changes Transient ST-segment elevation GRACE risk score > 140 (Class IIa LoE A)	Shift from Class I to Class IIa, reflecting real-world challenges in rapid patients’ referral and lack of conclusive superiority of a routine early invasive strategy.
**Pre-treatment in STEMI**	A potent P2Y12 inhibitor (prasugrel or ticagrelor), or clopidogrel if these are not available or are contraindicated, is recommended before (or at latest at the time of) PCI, and maintained over 12 months, unless there are contraindications such as excessive risk of bleeding (Class I LoE A) [2]	Pre-treatment with a P2Y12 receptor inhibitor may be considered in patients undergoing a primary PCI strategy (Class IIb LoE B)	Pre-treatment of STEMI may be considered but should not represent a routine approach.
**Multivessel Treatment Strategies**	(A) Routine immediate revascularization of non-culprit lesions in NSTE-ACS patients with multivessel disease presenting with CS is not recommended (Class III LoE B) [3]	(A) Staged PCI of non-IRA should be considered (Class IIa LoE C)	Upgrade to Class I, aligning with evidence from recent trials, with the exception of a cardiogenic shock presentation.
	(B) Routine revascularization of non-IRA lesions should be considered in STEMI patients with multivessel disease before hospital discharge (Class IIa LoE A) [2]	(B) Complete revascularization is recommended either during the index PCI procedure or within 45 days (Class I LoE A)	
	(C) Complete revascularization during index PCI may be considered in NSTE-ACS patients with multi- vessel disease (Class IIb LoE B) [3]	(C) In patients presenting with NSTE-ACS and MVD, complete revascularization should be considered, preferably during the index procedure (Class IIa LoE C)	
**Anatomy vs. Physiology for Non-Culprit Lesions in STEMI**	No clear indication of how to guide non-IRA lesions’ revascularization	It is recommended that the PCI of the non-IRA is based on angiographic severity (Class I LoE B)	Decision mostly driven by angiographic severity. Not recommended using FFR during index STEMI procedure.
**Role of non-invasive Imaging**	In patients with no recurrence of chest pain, normal ECG findings, and normal levels of cardiac troponin (preferably high sensitivity), but still with suspected ACS, a non-invasive stress test (preferably with imaging) for inducible ischaemia or CCTA is recommended before deciding on an invasive approach (Class I LoE B) [3]	In patients with suspected ACS, non-elevated (or uncertain) hs-cTn, no ECG changes and no recurrence of pain, incorporating CCTA or a non-invasive stress imaging test as part of the initial workup should be considered (Class IIa LoE A) #	Non-invasive imaging is no more recommended on a routine base, but should be considered in patients with a dubious presentation.
**Role of intracoronary imaging**	Intracoronary imaging should be considered to diagnose SCAD if suspected (Class IIa LoE C) [3]	Intravascular imaging should be considered to guide PCI (Class IIa LoE A) §	No change in class of recommendation but broader application of intracoronary imaging to guide PCI. This reflects the recent influx of data demonstrating significant improvements in patient outcomes when intracoronary imaging guides PCI.
**Antiplatelet Therapy Modulation**	A P2Y12 receptor inhibitor is recommended in addition to aspirin and maintained over 12 months unless there are contraindications or an excessive risk of bleeding (Class I LoE A) [3]	In all ACS patients, a P2Y12 receptor inhibitor is recommended in addition to aspirin, given as an initial oral LD followed by an MD for 12 months unless there is HBR (Class I LoE A) *	No change in recommendations irrespective of the accumulating evidence of shorter DAPT especially in HBR. A standardized approach is advised, which should or may be nuanced based on patients’ characteristics. No recommendations for a routine treatment personalization.
**Colchicine**	Not prominently featured	Low-dose colchicine (0.5 mg once a day) may be considered, particularly if other risk factors are insufficiently controlled or if recurrent cardiovascular disease events occur under optimal therapy (Class IIb LoE A)	New recommendation for anti-inflammatory agents to target secondary prevention.
**Adherence boosting strategies and polypill**	Not prominently featured	A polypill should be considered as an option to improve adherence and outcomes in secondary prevention after ACS (Class IIa LoE B)	New recommendation for the implementation of a polypill, with more attention to adherence-boosting strategies.

ACS: acute coronary syndrome; CCTA: Coronary Computed Tomography Angiography; FFR: Fractional Flow Reserve; GRACE: Global Registry of Acute Coronary Events; HBR: High Bleeding Risk; hs-cTn: High-Sensitivity Cardiac Troponin; IRA: Infarct-Related Artery; LD: loading dose; LoE: level of evidence; MD: maintenance dose; NSTE-ACS: Non-ST-elevation acute coronary syndrome; NSTEMI: Non-ST-elevation myocardial infarction; PCI: percutaneous coronary intervention; STEMI: ST-elevation myocardial infarction. # In patients with a working diagnosis of MINOCA cardiac magnetic resonance (CMR) imaging is recommended after invasive angiography if the final diagnosis is not clear (Class I LoE B). § Intravascular imaging (preferably optical coherence tomography) may be considered in patients with ambiguous culprit lesions. (Class IIb LoE C). * Deviations (i.e., abbreviated DAPT or DAPT de-escalation) from this default strategy could be adopted based on clinical scenario (see the text).

## Data Availability

Not applicable.
